# Lipidomics for the Prediction of Progressive Liver Disease in Patients with Alcohol Use Disorder

**DOI:** 10.3390/metabo12050433

**Published:** 2022-05-11

**Authors:** Bei Gao, Suling Zeng, Luca Maccioni, Xiaochun Shi, Aaron Armando, Oswald Quehenberger, Xinlian Zhang, Peter Stärkel, Bernd Schnabl

**Affiliations:** 1 School of Marine Sciences, Nanjing University of Information Science and Technology, Nanjing 210044, China; wintergb@hotmail.com; 2 Department of Medicine, University of California San Diego, La Jolla, CA 92093, USA; szeng@health.ucsd.edu (S.Z.); aarmando@health.ucsd.edu (A.A.); oquehenberger@health.ucsd.edu (O.Q.); 3 Laboratory of Hepato-Gastroenterology, Institute of Experimental and Clinical Research, Université Catholique de Louvain, 1200 Brussels, Belgium; luca.maccioni@uclouvain.be; 4 School of Environmental Science and Engineering, Nanjing University of Information Science and Technology, Nanjing 210044, China; 20201248131@nuist.edu.cn; 5 Division of Biostatistics and Bioinformatics, Department of Family Medicine and Public Health, University of California San Diego, La Jolla, CA 92093, USA; xizhang@health.ucsd.edu; 6 St. Luc University Hospital, Université Catholique de Louvain, 1200 Brussels, Belgium; 7 Department of Medicine, VA San Diego Healthcare System, San Diego, CA 92161, USA

**Keywords:** random forest, EPA, sphingomyelin, steatosis, alcohol-associated liver disease

## Abstract

Alcohol-related liver disease is a public health care burden globally. Only 10–20% of patients with alcohol use disorder have progressive liver disease. This study aimed to identify lipid biomarkers for the early identification of progressive alcohol-related liver disease, which is a key step for early intervention. We performed untargeted lipidomics analysis in serum and fecal samples for a cohort of 49 subjects, including 17 non-alcoholic controls, 16 patients with non-progressive alcohol-related liver disease, and 16 patients with progressive alcohol-related liver disease. The serum and fecal lipidome profiles in the two patient groups were different from that in the controls. Nine lipid biomarkers were identified that were significantly different between patients with progressive liver disease and patients with non-progressive liver disease in both serum and fecal samples. We further built a random forest model to predict progressive alcohol-related liver disease using nine lipid biomarkers. Fecal lipids performed better (Area Under the Curve, AUC = 0.90) than serum lipids (AUC = 0.79). The lipid biomarkers identified are promising candidates for the early identification of progressive alcohol-related liver disease.

## 1. Introduction

Chronic alcohol consumption induces steatosis through the acceleration of hepatic lipogenesis, deceleration of lipid breakdown, and defective export of hepatic lipids [1]. The majority of patients with alcohol use disorder develops hepatic steatosis. Alcohol-related steatosis is reversible upon cessation of alcohol consumption. However, only 10–20% of patients with heavy and daily drinking will develop progressive liver disease and cirrhosis. Various modifiers affect the progression of alcohol-related liver disease, such as the pattern of alcohol consumption, gender, age, race, genetics, nutritional factors, drugs, obesity, smoking, and viral infections [1]. Although the progression of alcohol-related liver disease is generally characterized from the histological point of view, the mechanisms that underlie the histological progression are still not well understood. In addition, there is currently no early prediction marker available to identify those who will develop liver fibrosis or cirrhosis. The ability to distinguish progressive liver disease from non-progressive liver disease would allow for the early and aggressive treatment of the underlying alcohol use disorder, which is an integral part for therapy of alcohol-related liver disease.

As a common hepatic change due to alcohol abuse, steatosis can invoke metabolic changes. The liver plays a key role in lipid metabolism, such as taking up fatty acids from dietary intake or the synthesis of fatty acids, which can be used as energy sources through β-oxidation or substrates for triglyceride biosynthesis in hepatocytes [2]. Fatty acid β-oxidation is inhibited by ethanol, increasing the availability of long-chain fatty acids and enhancing their esterification [3]. Dysregulation of lipid influx and efflux induced by ethanol further leads to the accumulation of lipids in hepatocytes. 

Tracer studies demonstrated that alcohol consumption induces alterations in de novo lipogenesis [4,5]. Chronic alcohol consumption increased hepatic free fatty acids, ceramide metabolites, and decreased the level of acyl-CoA in a mouse model [6]. In addition, tissue-specific changes in fatty acids induced by ethanol were also reported in a mouse model [7]. Dysregulation of the serum lipid profile was found in a patient cohort of 59 excessive alcohol drinkers [8]. Although the serum lipidome has been studied to some extent, the fecal lipidome is not well characterized in patients with alcohol use disorder. It is not clear whether fecal lipids can be used to predict progressive alcohol-related liver disease. In addition, fecal samples present an advantage over serum samples, as they can be collected in a non-invasive way.

In the present study, we performed a serum and fecal lipidomics analysis in a cohort of 49 subjects, including non-alcoholic controls and patients with non-progressive and progressive alcohol-related liver disease. We aimed to: (1) reveal the alteration of serum and fecal lipids in two patient groups compared with control subjects; (2) identify serum and fecal lipid biomarkers that distinguish between progressive and non-progressive alcohol use disorder; and (3) build a random forest model to predict progressive alcohol-related liver disease.

## 2. Results

### 2.1. Patient Cohort

A total of 17 non-alcoholic controls, 16 patients with non-progressive alcohol-related liver disease, and 16 patients with progressive alcohol-related liver disease, based on our clinical classification, were included in this study. CK-18-M65 supported this classification, with 33% of patients in the non-progressive, and 93% in the progressive, alcohol-related liver disease group showing levels below or above 400 U/L, respectively. Patient characteristics are summarized in Table 1. Patients with steato-hepatitis or steato-fibrosis had a preserved synthetic liver function and showed no clinical signs of liver decompensation. There was no significant difference in age, body mass index, and gender between control subjects and the two patient groups. Compared with patients with non-progressive liver disease, patients with progressive liver disease showed significantly higher levels of ALT, AST, GGT, total bilirubin, CAP, and CK18-M65 (Table 1).

### 2.2. Serum Lipidome

**Figure 1:**
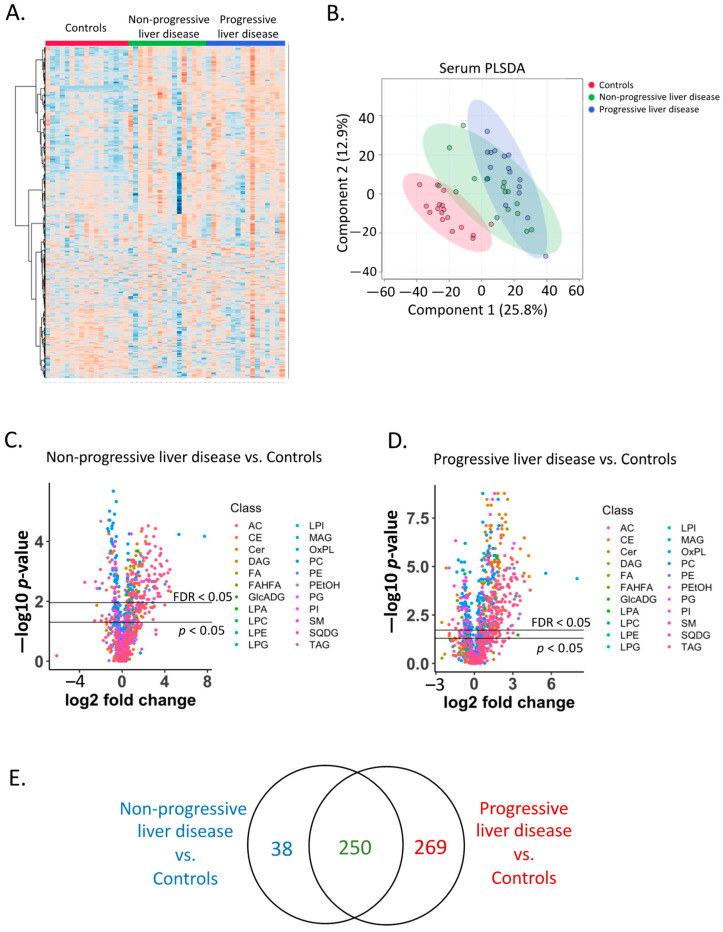
Serum lipidome. (**A**) Heatmap of the serum lipidome in the control and two patient groups. (**B**) Partial least squares-discriminant analysis of the serum lipidome. (**C**) Volcano plot of the serum lipidome in control subjects and patients with non-progressive liver disease. Different colors represent different lipid classes. Fold change: non-progressive liver disease (*n* = 16)/controls (*n* = 17). (**D**) Volcano plot of the serum lipidome in control subjects and patients with progressive liver disease. Different colors represent different lipid classes. Fold change: progressive liver disease (*n* = 16)/controls (*n* = 17). (**E**) Venn diagram of significant serum lipids (adjusted *p*-value < 0.05). Dark blue: number of unique significant lipids between non-progressive liver disease and controls; Red: number of unique significant lipids between progressive liver disease and controls; Dark green: shared significant lipids.

Hierarchical clustering of the serum lipidome showed the profile of serum lipids from control subjects was different from patients with non-progressive and progressive liver disease (Figure 1A, Supplementary File). A partial least square discriminant analysis (PLSDA) showed that control groups were separated from the two patient groups (Figure 1B). A Kruskal–Wallis rank sum test for component 1 showed a *p*-value of 1.2 × 10^−6^ between three groups (Nemenyi-tests for pairwise comparison between non-progressive liver disease vs. controls *p*-value = 0.0006; progressive liver disease vs. controls *p*-value = 1.6 × 10^−6^; progressive liver disease vs. non-progressive liver disease *p*-value = 0.41). For component 2, the comparison between the three groups showed a *p*-value of 0.002 (Nemenyi-tests for pairwise comparison between non-progressive liver disease vs. controls *p*-value = 0.038; progressive liver disease vs. controls *p*-value = 0.0019; progressive liver disease vs. non-progressive liver disease *p*-value = 0.61). 

Out of 1266 annotated serum lipids, 481 showed a *p*-value < 0.05 and 288 showed an adjusted *p*-value < 0.05 when comparing patients with non-progressive liver disease with controls (Figure 1C). When comparing patients with progressive liver disease with controls, 640 serum lipids showed a *p*-value < 0.05 and 519 showed an adjusted *p*-value < 0.05 (Figure 1D). A total of 250 serum lipids were found to be significantly different (adjusted *p*-value < 0.05) in both patients with non-progressive liver disease and in patients with progressive liver disease compared with controls (Figure 1E). Compared with control subjects, 38 serum lipids were significantly different (adjusted *p*-value < 0.05) only in patients with non-progressive liver disease; meanwhile, 269 serum lipids were significantly different (adjusted *p*-value < 0.05) only in patients with progressive liver disease (Figure 1E).

### 2.3. Fecal Lipidome

**Figure 2:**
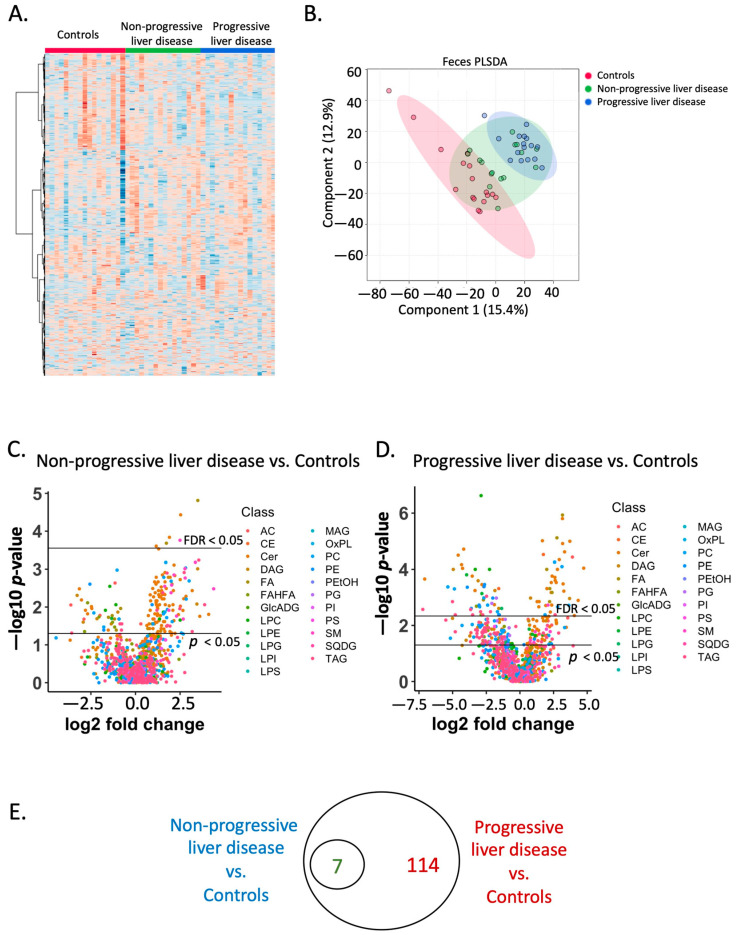
Fecal lipidome. (**A**) Heatmap of the fecal lipidome in the control and two patient groups. (**B**) Partial least squares-discriminant analysis of the fecal lipidome. (**C**) Volcano plot of the fecal lipidome in control subjects and patients with non-progressive liver disease. Different colors represent different lipid classes. Fold change: non-progressive liver disease (*n* = 16)/controls (*n* = 17). (**D**) Volcano plot of the fecal lipidome in control subjects and patients with progressive liver disease. Different colors represent different lipid class. Fold change: progressive liver disease (*n* = 16)/controls (*n* = 17). (**E**) Venn diagram of significant fecal lipids (adjusted *p*-value < 0.05). Red: number of unique, significant lipids between progressive liver disease and controls; Dark green: shared significant lipids.

Hierarchical clustering of the fecal lipidome showed that the profile of fecal lipids from control subjects was different from the two patient groups (Figure 2A, Supplementary File). A PLSDA plot showed control groups were generally separated from the two patient groups (Figure 2B). A Kruskal–Wallis rank sum test for component 1 showed a *p*-value of 5.7 × 10^−7^ between the three groups (Nemenyi-tests for pairwise comparison between non-progressive liver disease vs. controls *p*-value = 0.0081; progressive liver disease vs. controls *p*-value = 2.7 × 10^−7^; progressive liver disease vs. non-progressive liver disease *p*-value = 0.052). For component 2, a comparison between the three groups showed a *p*-value of 0.0008 (Nemenyi-tests for pairwise comparison between non-progressive liver disease vs. controls *p*-value = 0.24; progressive liver disease vs. controls *p*-value = 0.00048; progressive liver disease vs. non-progressive liver disease *p*-value = 0.087). 

Out of 1219 fecal lipids, 218 showed a *p*-value less than 0.05 and seven showed an adjusted *p*-value less than 0.05 when comparing patients with non-progressive liver disease with controls (Figure 2C). When comparing patients with progressive liver disease with controls, 357 fecal lipids showed a *p*-value < 0.05 and 121 showed an adjusted *p*-value less than 0.05 (Figure 1D). Compared with controls, seven fecal lipids were found to be significantly different (adjusted *p*-value < 0.05) in both patients with non-progressive liver disease and in patients with progressive liver disease; meanwhile, 114 fecal lipids were found to be significantly different (adjusted *p*-value < 0.05) only in patients with progressive liver disease (Figure 2E).

### 2.4. Lipid Biomarkers for Progressive Liver Disease

**Figure 3:**
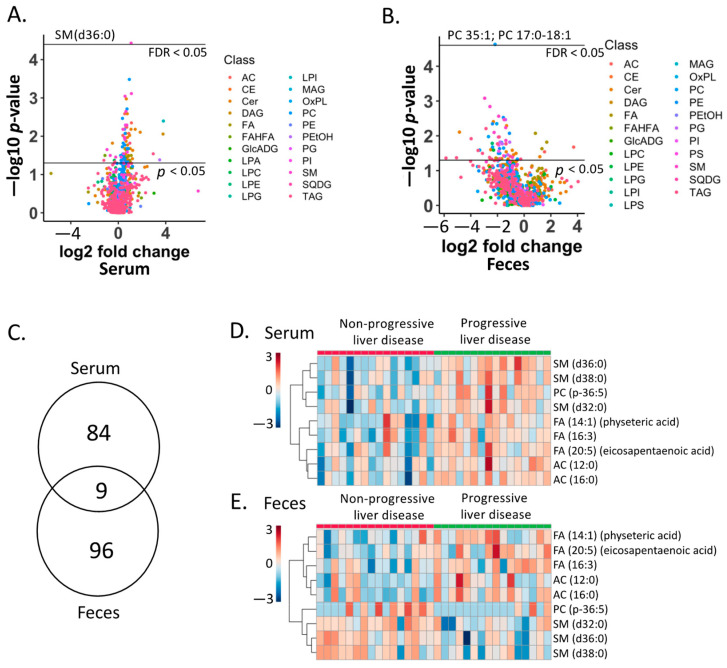
Comparison between progressive liver disease and non-progressive liver disease. (**A**) Volcano plot of the serum lipidome for the two patient groups. Different colors represent different lipid class. Fold change: progressive liver disease (*n* = 16)/non-progressive liver disease (*n* = 16). (**B**) Volcano plot of the fecal lipidome for the two patient groups. Different colors represent different lipid class. Fold change: progressive liver disease (*n* = 16)/non-progressive liver disease (*n* = 16). (**C**) Venn diagram of significant fecal lipids (*p*-value < 0.05). (**D**) Heatmap of nine serum lipid biomarkers. (**E**) Heatmap of nine fecal lipid biomarkers. SM: sphingomyelin; PC: phosphatidylcholine; FA: fatty acid; AC: acylcarnitine.

In order to find the biomarkers that can distinguish progressive liver disease from non-progressive liver disease, we compared serum and fecal lipids in the two patient groups. A total of 93 serum lipids showed *p*-values < 0.05, with 10 decreased in progressive liver disease and 83 increased in progressive liver disease compared with non-progressive liver disease (Figure 3A). One serum lipid, SM (d36:0), was significantly increased in progressive liver disease, with an adjusted *p*-value < 0.05 (Figure 3A). A total of 106 fecal lipids showed *p*-values < 0.05, with 96 decreased and 10 increased in progressive liver disease compared with non-progressive liver disease (Figure 3B). One fecal lipid, PC 35:1; PC17:0-18:1, was significantly increased in progressive liver disease with an adjusted *p*-value < 0.05 (Figure 3B). Notably, nine lipids were found to be significantly different in both the serum and feces (*p*-value < 0.05) (Figure 3C). These lipids were increased in the serum of patients with progressive liver disease compared with non-progressive liver disease (Figure 3D). In the fecal samples, five out of nine lipids were increased; meanwhile, the remaining four lipids were decreased in patients with progressive liver disease compared with non-progressive liver disease (Figure 3E). 

### 2.5. Association of Lipid Biomarkers with Clinical Parameters

**Figure 4:**
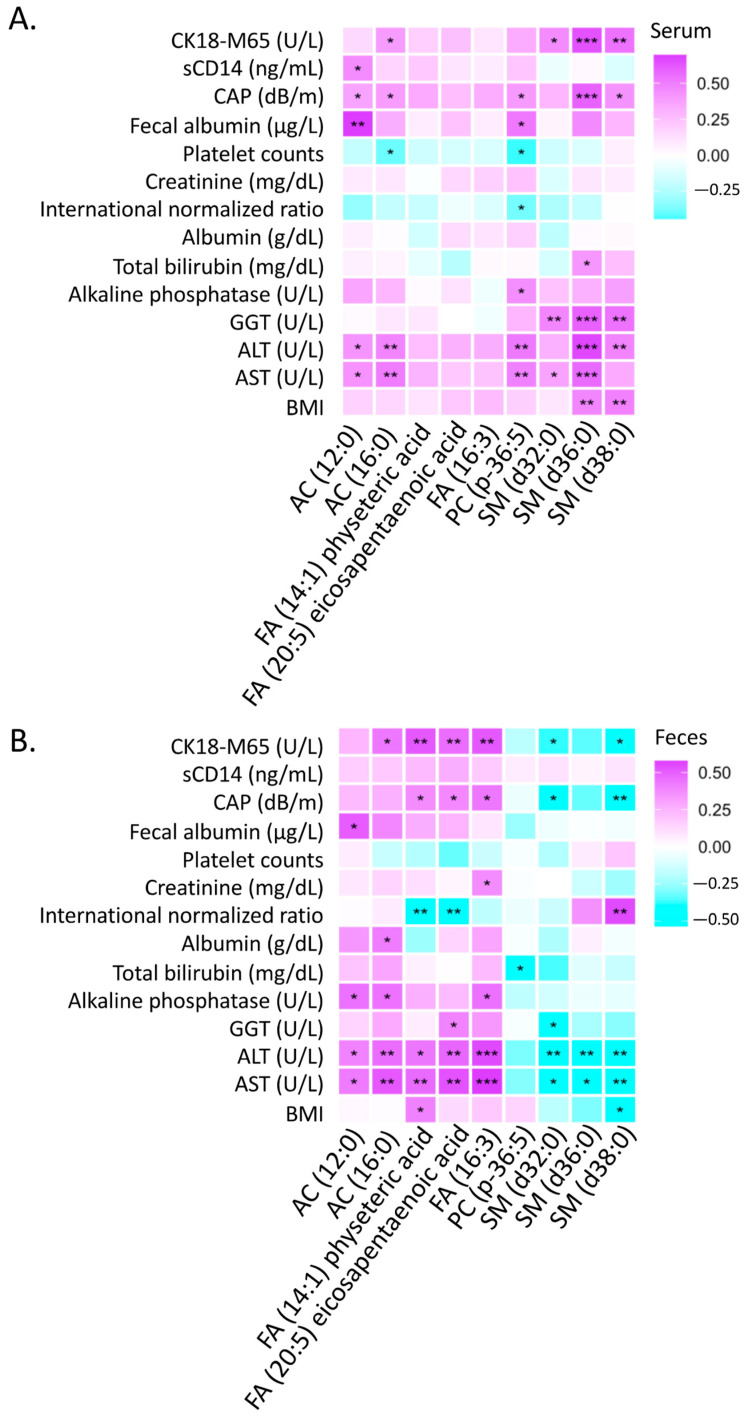
Spearman correlation between nine lipid biomarkers and clinical parameters. (**A**) Serum lipids. (**B**) Fecal lipids. *: *p*-value < 0.05; **: *p*-value < 0.01; ***: *p*-value < 0.001.

We further examined the association of the nine lipid biomarkers with 14 clinical parameters. A Spearman correlation analysis showed that acylcarnitines, phosphatidylcholine, and sphingomyelins in the serum samples correlated with multiple clinical parameters, including BMI, AST, ALT, GGT, alkaline phosphatase, total bilirubin, international normalized ratio, platelet counts, fecal albumin, CAP, sCD14, and CK18-M65 (Figure 4A). However, fatty acids in the serum samples did not correlate with any clinical parameters (Figure 4A). In contrast, all of the nine lipid biomarkers in the fecal samples, including fatty acids, were significantly correlated with multiple clinical parameters (Figure 4B).

### 2.6. Prediction of Progressive Liver Disease

**Figure 5:**
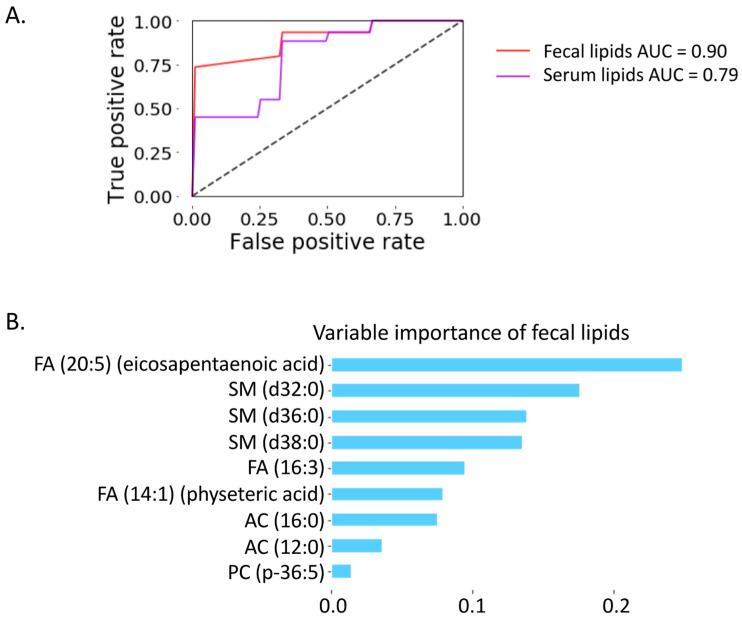
Prediction of progressive liver disease using nine lipid biomarkers. (**A**) Random forest model. Red: nine fecal lipids AUC = 0.90; Purple: nine serum lipids AUC = 0.79. (**B**) Variable importance.

To distinguish progressive liver disease from non-progressive liver disease, we built a random forest model using the identified nine lipid biomarkers (Figure S1). The area under the curve achieved 0.79 when using nine serum lipids as variables (Figure 5A). Interestingly, AUC achieved 0.90 when using nine fecal lipids as variables (Figure 5A). Among the nine fecal lipids, the most important variable was eicosapentaenoic acid (EPA) (Figure 5B), which was significantly increased (*p*-value < 0.05) in serum and feces from patients with progressive liver disease compared with non-progressive liver disease (Figure 3D,E). The fecal level of EPA was positively correlated with AST, ALT, GGT, CAP, and CK18-M65, and negatively correlated with the international normalized ratio (Figure 4B).

### 2.7. Microbial Lipid Pathways

**Figure 6:**
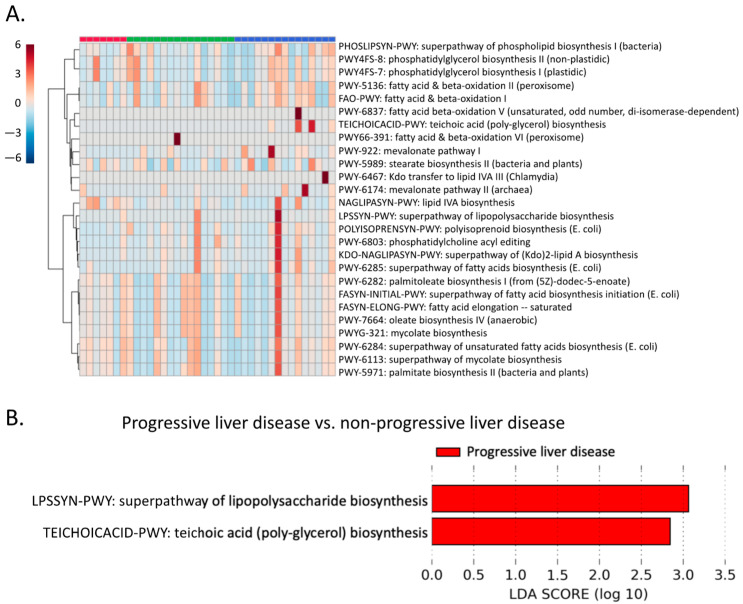
Microbial lipid metabolism. (**A**) Fecal microbial lipid pathways detected using metagenomic sequencing of fecal DNA. (**B**) Enriched fecal microbial lipid pathways in patients with progressive liver disease compared with non-progressive liver disease. LDA: Linear Discriminant Analysis.

Given that nine fecal lipids showed better predictive power than serum lipids, we further investigated the potential contribution of gut microbiota to the host fecal lipidome. A total of 26 microbial lipid pathways were detected by shotgun metagenomic analysis (Figure 6A). A Lefse analysis showed that two microbial pathways were enriched in patients with progressive liver disease compared with non-progressive liver disease, including the LPSSYN-PWY superpathway of lipopolysaccharide biosynthesis and TEICHOICACID-PWY teichoic acid (poly-glycerol) biosynthesis (Figure 6B).

## 3. Discussion

Although alcohol-related steatosis is generally considered as rather benign and reversible, it can further develop into steatohepatitis with fibrosis and progression to cirrhosis. The identification of effective and non-invasive biomarkers for progressive liver disease as early as possible during the disease course is important as it likely impacts patient management. In our present study, we identified lipid biomarkers in both fecal and serum samples. Interestingly, fecal lipids performed better than serum lipids to predict progressive liver disease (Figure 5A). Since fecal samples can be collected in a non-invasive way, they can serve as an alternative for the currently widely used serum biomarkers. It is noteworthy that the changing trajectory of fecal lipids could not fully represent the changing trajectory of circulating lipids. As shown in Figure 3D,E, although the changing direction for five lipids was consistent in both serum and fecal samples, including FA (14:1), FA (20:5), FA (16:3), AC (12:0), and AC (16:0), the other four lipids showed an opposite changing direction in serum and fecal samples. 

As an omega-3 polyunsaturated fatty acid, EPA was the most important variable in our random forest model to predict progressive liver disease (Figure 5B). An increased level of EPA was found in both serum and fecal samples of patients with progressive liver disease compared with non-progressive liver disease (Figure 3D,E). The impact of EPA on alcohol-related liver disease is not well-understood. However, in non-alcoholic fatty liver disease, supplementation of EPA at 1.8 or 2.8 g/L showed no benefit in blood or hepatic markers of non-alcoholic steatohepatitis in a phase II clinical trial with 243 subjects enrolled at 37 sites in North America [9]. 

Three sphingomyelin species were increased in serum samples, while they were decreased in the fecal samples of patients with progressive liver disease compared with non-progressive liver disease (Figure 3D,E). Sphingolipids are ubiquitous constituents of the cell membrane. Hydrolysis of membrane sphingomyelins by acid sphingomyelinases produces ceramides, which serve as a second messenger in the sphingomyelin signaling pathway and regulate a wide range of cellular responses [10]. Acid sphingomyelinases could be activated by alcohol, which has been proven to be a regulator of steatosis, fibrosis, lipotoxicity, and endoplasmic reticulum stress [11]. 

Although lipid metabolism is mainly controlled by the host, intestinal microbes also affect host lipid composition, including the production of sphingolipids, polyunsaturated fatty acid-derived metabolites, and hydroxy fatty acids [12,13,14,15]. Bacterial biosynthesis of saturated long chain fatty acids was reduced by chronic ethanol feeding in a mouse model [16]. In the present study, we found that two microbial lipid pathways were enriched in progressive liver disease compared with non-progressive liver disease, including lipopolysaccharide biosynthesis and teichoic acid (poly-glycerol) biosynthesis (Figure 6B). An in vitro study in primary hepatocytes showed that lipopolysaccharide potentiated the effects of the ethanol on the sphingomyelin cycle [17]. In addition, lipopolysaccharide is an important mediator of alcohol-related liver disease. Bacterial lipopolysaccharide translocates to the liver, which is facilitated by a disrupted gut barrier. Lipopolysaccharide binds to its receptor Toll-like receptor 4 in the liver, which contributes to the progression of alcohol-related disease [18]. Thus, increased microbial lipopolysaccharide biosynthesis could contribute to disease progression. In addition to lipopolysaccharide, which is found in the outer membrane of Gram-negative bacteria, the biosynthesis of teichoic acids was also increased, which is mostly found within the cell wall of Gram-positive bacteria (Figure 6B). Teichoic acids include both lipoteichoic acids and wall teichoic acids. Lipoteichoic acids are the agonist of Toll-like receptor 2 [19]. The activation of Toll-like receptor 2 also contributes to alcohol-related liver disease [20].

The limitations of this study include that the sample size is relatively small, the information on polymorphism, alcohol dehydrogenase (ADH), or Cytochrome P450 2E1 enzymes in patients is not available; liver tissue in this patient cohort is not available either. Although we used a cross-validation approach in the random forest model, our findings need to be confirmed in an independent, larger patient cohort using targeted lipid analysis. Despite this, we identified that lipid biomarkers, especially fecal lipids, showed great power to predict progressive liver disease at an early stage. This will allow physicians to initiate aggressive treatment of the underlying alcohol use disorder, which is currently the most important intervention for alcohol-related liver disease.

## 4. Materials and Methods

### 4.1. Patients

A total of 32 alcohol use disorder (AUD) patients (Diagnostic and Statistical Manual of Mental Disorders, Fourth Edition criteria) admitted for elective alcohol withdrawal to a dedicated alcohol withdrawal unit were recruited for the study. Inclusion and exclusion criteria has been described in our previous study [21]. Stool samples were collected from the first bowel movement after admission. They were compared to a total of 17 nonalcoholic controls, who were social drinkers and consumed less than 20 g of alcohol per day, matched for gender, age, and BMI. During the two months preceding enrollment, nonalcoholic controls or patients with alcohol use disorder did not take immunosuppressive medication or antibiotics.

AUD patients were split into non-progressive liver disease and progressive liver disease based on clinical parameters as described in our previous publication [21]. The determination of blood cytokeratin 18, soluble CD14, and fecal albumin levels has been described in our previous study [21].

### 4.2. Lipidomics Analysis

Serum and fecal sample extraction was performed as described in our previous publication [22]. Fecal samples (10 mg) were homogenized at 1500 rpm for 30 s using a GenoGrinder 2010 (SPEX SamplePrep, Metuchen, NJ, USA). Serum samples (20 μL) were extracted without homogenization. Lipidomics data acquisition was performed as described in our previous publication [22]. LC-MS raw data files were converted to ABF files using ABF converter (https://www.reifycs.com/AbfConverter/, accessed on 4 March 2021) and then processed by MS-DIAL version 2.94 [23] and MS-FLO [24] as described in our previous publication [22]. For compound identification, retention time-*m*/*z* libraries and MS/MS spectra databases were used as uploaded to MassBank of North America. Features present in at least 50% of samples in each group were reported. 

### 4.3. Shotgun Metagenomics Analysis

Due to the sample availability, fecal DNA was extracted from stool samples in seven control subjects, 16 patients with non-progressive liver disease, and 15 patients with progressive liver disease using a FastDNA Spin Kit for Soil (MP-Biomedicals, Irvine, CA, USA) as described in our previous publication [25]. Illumina HiSeq 4000 generating 150 bp paired-end reads was used for shotgun metagenomics sequencing. Quality control of reads was performed using KneadData version 0.7.2 (https://huttenhower.sph.harvard.edu/kneaddata/, accessed on 4 March 2021), followed by The HMP Unified Metabolic Analysis Network 2 (HUMAnN2) version 0.11.1 (https://huttenhower.sph.harvard.edu/humann2/, accessed on 4 March 2021) for the profiling of microbial pathways [26]. MetaCyc database was used for the fecal microbial pathway analysis [27]. Each of the HUMAnN2 abundance output was normalized into relative abundance. A linear discriminant analysis (LDA) effect size (LEfSe) was used in this study for the biomarker discovery [28]. 

### 4.4. Statistical Analysis

R (version 3.6.2) (https://cran.r-project.org/bin/windows/base/old/3.6.2/, accessed on 4 March 2021) was used for statistical analysis. A Kruskal–Wallis and Mann–Whitney–Wilcoxon test was used for the comparison of three groups and two groups, respectively. The Benjamini–Hochberg procedure was used to control the false discovery rate. Heatmap and PLS-DA plots were generated using MetaboAnalyst 4.0 [29]. The Spearman correlation was conducted to correlate clinical parameters with serum or fecal lipids. A random forest model was built on the H_2_O platform (https://www.h2o.ai, accessed on 6 April 2021) to predict the progressive liver disease using serum and fecal lipids. The lipidomics dataset was split into training and test datasets (80:20 stratified splits). We performed a stratified 5-fold cross-validation on the training set to choose the tuning parameters for the model.

## Figures and Tables

**Table 1:** Subject characteristics.

	Non-Alcoholic Controls	Non-Progressive Liver Disease	Progressive Liver Disease	*p*-Value
Clinical parameter				
Total *n*	17	16	16	
Age, years, *n* = 48	38 (27–71)	37 (27–58)	41 (28–59)	0.470
Body Mass Index (BMI), kg/m², *n* = 48	22 (19–29)	22 (19-31)	24 (18–31)	0.381
Gender (male), *n* (%), *n* = 48	14 (88)	11 (69)	14 (88)	0.292
Laboratory parameter				
Albumin (g/dL), *n* = 27		4.7 (4.2–5.2)	4.7 (3.9–5.2)	0.558
Alkaline phosphatase (U/L), *n* = 28		65 (38–101)	81 (47–113)	0.072
ALT (U/L), *n* = 32		19 (11–37)	78 (37–184)	<0.001
AST (U/L), *n* = 32		25 (15–36)	81 (46–283)	<0.001
Total bilirubin (mg/dL), *n* = 29		0.3 (0.2–1.1)	0.5 (0.3–0.9)	0.031
GGT (U/L), *n* = 29		31 (4–213)	139 (11–952)	0.012
Platelet counts (×10^9^/L), *n* = 28		268 (165–339)	220 (21–434)	0.270
Creatinine (mg/dL), *n* = 29		0.8 (0.5–1.0)	0.8 (0.6–1.2)	0.406
International normalized ratio, *n* = 29		1.0 (0.9–1.2)	0.9 (0.8–1.0)	0.115
Fibroscan (kpa), *n* = 32		4.8 (3.1–6.6)	6.0 (3.2–7.0)	0.122
CAP, (dB/m), *n* = 32 CAP > 250 dB/m, *n* (%)		254 (148–325) 9 (56)	314 (222–381) 15 (94)	<0.001
Fecal albumin (µg/L), *n* = 43	16.9 (4.7–66.6)	56.8 (10.5–504.4)	31.2 (2.2–98.1)	0.002
CK18-M65 (U/L), *n* = 38	166 (104–282)	332 (158–616)	592 (316–1576)	<0.001
sCD14 (ng/mL), *n* = 38	1376 (1074–1810)	1710 (1046–2570)	1745 (1191–2266)	0.033

Values are presented as medians with ranges in parentheses (·). ALT, alanine aminotransferase; AST, aspartate aminotransferase; GGT, gamma-glutamyl-transferase; CAP: controlled attenuation parameter. Fecal albumin: Non-progressive liver disease vs. controls: *p*-value < 0.001; Progressive liver disease vs. controls: *p*-value = 0.209; Progressive liver disease vs. Non-progressive liver disease: *p*-value = 0.118. CK18-M65: Non-progressive liver disease vs. controls: *p*-value = 0.084; Progressive liver disease vs. controls: *p*-value < 0.001; Progressive liver disease vs. Non-progressive liver disease: *p*-value = 0.006. sCD14: Non-progressive liver disease vs. controls: *p*-value = 0.053; Progressive liver disease vs. controls: *p*-value = 0.040; Progressive liver disease vs. Non-progressive liver disease: *p*-value = 0.991.

## Data Availability

Data is available upon request. Contact information: Bernd Schnabl, (M.D., Department of Medicine, University of California San Diego, Email beschnabl@health.ucsd.edu). The data are not publicly available due to privacy restrictions.

## Supplementary Materials

The following supporting information can be downloaded at: https://www.mdpi.com/article/10.3390/metabo12050433/s1, Figure S1: Boxplot of nine fecal lipids used for the prediction of progressive liver disease. Supplementary File: Serum and fecal lipidome.
