# Supplementary material for: Lipidomics for the Prediction of Progressive Liver Disease in Patients with Alcohol Use Disorder
Source: Metabolites. 2022 May 11;12(5):433. doi: 10.3390/metabo12050433 (PMC9146183; doi:10.3390/metabo12050433)
Supplement: Supplementary file 1 [file metabolites-12-00433-s001.zip › metabolites-1713371-Figure S1.pdf]

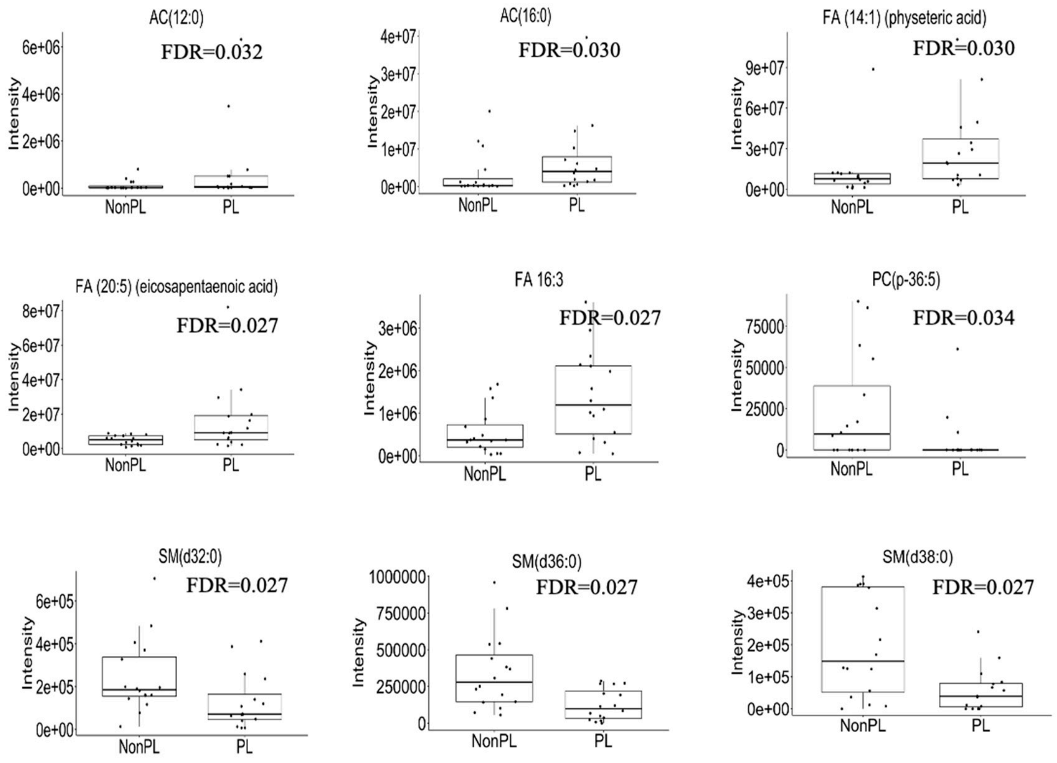

Figure S1. Boxplot of nine fecal lipids used for the prediction of progressive liver disease. NonPL: Patients with non-progressive liver disease; PL: Patients with progressive liver disease.
